# Liver Zonation in Health and Disease: Hypoxia and Hypoxia-Inducible Transcription Factors as Concert Masters

**DOI:** 10.3390/ijms20092347

**Published:** 2019-05-11

**Authors:** Thomas Kietzmann

**Affiliations:** Faculty of Biochemistry and Molecular Medicine, Biocenter Oulu, University of Oulu, 90220 Oulu, Finland; Thomas.Kietzmann@oulu.fi; Tel.: +358-294487713

**Keywords:** ROS, antioxidants, antioxidative enzymes, matrix, diet, fibrosis, homeostasis, hypoxia, HIF, liver, metabolic zonation, metabolism, morphogen signaling, optimization, pathology, regulatory network, sinusoid, Hepatocytes

## Abstract

The liver and its zonation contribute to whole body homeostasis. Acute and chronic, not always liver, diseases impair proper metabolic zonation. Various underlying pathways, such as β-catenin, hedgehog signaling, and the Hippo pathway, along with the physiologically occurring oxygen gradient, appear to be contributors. Interestingly, hypoxia and hypoxia-inducible transcription factors can orchestrate those pathways. In the current review, we connect novel findings of liver zonation in health and disease and provide a view about the dynamic interplay between these different pathways and cell-types to drive liver zonation and systemic homeostasis.

## 1. Introduction

The liver is a central organ for maintaining systemic homeostasis. It acts as a center of energy supply in all metabolic states, as a center of defense when eliminating xenobiotic macromolecules, and as a center of erythropoiesis during embryonic life and later as a blood reservoir. The liver is also an important production site of hormones, growth factors, and, last but not least, the bile. Via vagal and splanchnic nerves, the liver also contributes to the control of food intake by the central nervous system. The action of parenchymal cells and four main types of nonparenchymal cells, either alone or in cooperation carry out all processes required for homeostasis maintenance. To achieve this in the most efficient manner, the liver parenchyma displays a so-called “metabolic zonation”. Due to the location within the circulation and the multitude of functions, the liver is susceptible to various diseases, which often compromise liver zonation. Therefore, the understanding of the pathways contributing to metabolic zonation could be crucial to combat certain disease pathologies. Various signal pathways have been shown to play a role in the regulation of zonation among them the oxygen-sensing system [1]. Given the importance of the presence of oxygen for cell survival, but also the production of either signaling or toxic reactive oxygen species (ROS), we concentrate here on the role of the oxygen-signaling pathway, which centers at the hypoxia-inducible factors (HIFs) for metabolic zonation. Thereby, we will connect recent observations on regulatory processes on zonation in health and disease and their potential use for therapeutic interventions.

## 2. Liver Lobule and Acinus—Structural and Functional Units Displaying Metabolic Zonation

The hexagonal lobule, with its portal triads at the corners and the central vein in the middle, represents the smallest structural unit of the liver; the acinus centered around a portal tract and extending to the respective neighboring central veins, thereby integrating the lobule, is considered to be the smallest unit in terms of function [2]. Blood enters the liver from two afferent vessels, the portal vein and the hepatic artery. Branches of the portal vein deliver nutrient and hormone rich blood to the portal tracts, whereas the hepatic artery provides oxygen-rich blood. The mixture of the blood flows then through the sinusoids and leaves the lobule or acinus via the central veins. As a result, the periportal oxygen content is about 60 to 65 mm Hg (84–91 µmol/L) and declines to about 30 to 35 mm Hg (42–49 µmol/L) in the perivenous area (for review see [1,3]). The sinusoids between the hepatocyte chords are key for maintaining systemic homeostasis. They mainly consist of highly fenestrated endothelial cells, and for defense purposes, they also contain Kupffer cells. The Space of Disse between the hepatocytes and the endothelial cells provides room for Stellate cells and contributes to draining of lymph. Overall, the structure of the sinusoids allows nutrients, metabolites, and various substrates to be freely exchanged between hepatocytes and blood [1,2].

**Figure 1:**
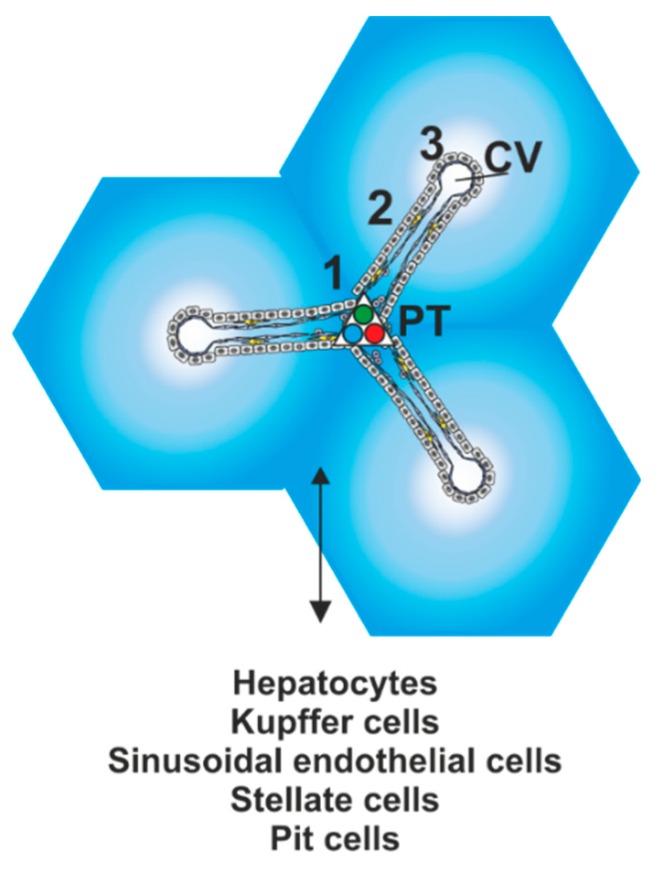
Microcirculatory architecture of the liver acinus. Classic hexagonal lobules are centered around a portal tract (PT) and blood flows as mixture of arterial and venous blood into the sinusoids which extend into the direction of the central veins (CV). The oxygen gradient generated ranges from ~60–65 mm Hg (dark blue) to ~30–35 mm HG (light blue); three zones exist: one around the portal triads (i.e., the periportal zone or zone 1), another around the central vein (i.e., the perivenous zone, or zone 3) and a midzonal layer or zone 2. Hepatocytes, fenestrated endothelial cells, hepatic stellate cells residing in the Space of Disse, separating hepatocytes and endothelial cells, Kupffer cells as resident macrophages and Pit cells are the building blocks of the sinusoids.

As a result, the liver lobules/acini show functional variance along the portal–central axis with respect to subcellular [4,5], biochemical, and physiological functions [6]. Accordingly, several zones can be distinguished from each other: one zone exists around the portal triads (i.e., the periportal zone or zone 1), another around the central vein (i.e., the perivenous zone, or zone 3); an interestingly active intermediary space (i.e., midzonal layer or zone 2) [7] exists in-between (Figure 1). 

Although not all functions need to display a zonation, all major metabolic pathways were found to be zonated and even integrated into mathematic models (for review see [1,2,3,4,5,6,7,8,9]). Moreover, nonparenchymal cell localization and functions are also zonated [10,11].

Apart from generating efficiency with respect to the action of metabolic pathways, zonation limits substrate competition and futile cycles. Further, it provides protection by linking complementing pathways and restricting injuries to zonal locations. Overall, this allows and integrates the powerful capacity of the liver to regenerate, to replace dead cells, and to restore its structure and function.

## 3. Regulatory Pathways Involved in Liver Zonation

As liver zonation is largely dynamic, it is regulated by a variety of signaling pathways as well as the interplay of the different cell types within the lobule/acinus. Several lines of evidence indicated that the Wnt/β-catenin pathway [12] and the associated R-spondin-leucine-rich repeated-containing G protein-coupled receptor 4/5 (LGR 4/5) axis [13], the extracellular signal-regulated kinase (RAS/ERK) pathway [14], the Hippo pathway with its cotranscriptional activator yes-associated protein 1 (Yap) [15], the hedgehog pathway [16], glucagon signaling [17], hepatocyte nuclear factor 4-α [18], Dicer [19], and the oxygen gradient contribute to hepatic zonation. All these pathways and factors, except the oxygen gradient, depend, to a greater or lesser extent, on the presence of specific ligands such as WNTs, or various growth factors, which are secreted from other cells than the hepatocytes (for review see [20]). For example, in mice, the liver endothelial cells contribute to the synthesis of Wnt2, Wnt9b, [21], and R-spondin 3 (RSPO3) [22], whereas cholangiocytes and stellate cells can secrete hedgehog ligands [23]. In addition to the large extent of cross-talk between all these pathways, the intercellular cooperation was further supported by the observation that angiocrine WNT signaling, particularly from sinusoidal endothelial cells, contributes to metabolic maturation and zonation of hepatocytes [24]. Considering the overlapping aspects and taking into account that the nature of the sinusoids facilitates free exchange not only of the above-mentioned factors, but also nutrients, metabolites, and various substrates, it is the periportal to perivenous oxygen concentration gradient that exists inevitably under all physiological and pathophysiological conditions, although the extent of the gradient may vary.

## 4. Oxygen Sensing and Hypoxia-Inducible Transcription Factors (HIFs)

The transcriptional adaptation in response to changing oxygen levels in a cell is mediated by the α-subunits of hypoxia-inducible factors (HIFα). Three HIFs (for review see [25,26]), showing a predominantly perivenous expression pattern in liver [27] have been identified so far. HIFα subunits form a complex with the beta subunit known as ARNT and bind to hypoxia responsive elements (HREs) in target genes [28]. Although the HIF-1α and HIF-2α-containing complexes share the HRE consensus, they were found to occupy a distinct set of genomic sites, which varied with cell type. In addition, lack of binding due to absence of the one or the other HIFα subunit could not be supplemented by the remaining HIFα variant [29]. Thus, these features may explain, at least partially, why HIF-1α was suggested to account for an acute and HIF-2α for a chronic response to hypoxia [30]. HIF-3α is far less explored. Several splice variants were identified [31] and their role in the hypoxia response is conflicting; some splice variants were shown to play a role as inhibitor of the hypoxia response [32,33], whereas other variants were identified as activators [34,35].

All HIFα subunits undergo proteasomal degradation under normoxic conditions. This is achieved by an intricate interplay of HIF prolyl 4-hydroxylases (commonly known as PHDs, EglNs or HIF-P4H), and an E3-ubiquitin ligase complex with the von Hippel Lindau (VHL) protein as substrate binding component. Like with HIFs, three HIF prolyl 4-hydroxylases have been identified; they act as cellular oxygen sensors and hydroxylate one or two proline residues within the HIFα subunits in an O_2_-dependent manner; these hydroxyprolines serve as docking site for the VHL protein [36]. Apart from O_2_, the reaction also requires 2-oxoglutarate (2-OG), Fe^2+^, and ascorbate. If oxygen becomes limited, the HIF prolyl 4-hydroxylases become inhibited and HIFs escape the proteasomal degradation. A similar O_2_-dependent enzymatic mechanism carried out by the asparagine hydroxylase factor-inhibiting HIF (FIH) prevents binding of the transcriptional coactivator CBP/p300 to the C-terminal transactivation domain (CTAD) of HIF-1α and HIF-2α. With respect to HIFs, this action is specific for HIF-1α and HIF-2α since even full-length HIF-3α lacks a CTAD (for review see [37] and references therein).

Hypoxia exerts a feedback and activates expression of HIF prolyl 4-hydroxylases-2 and -3 via HIFs; consequently, this rehydroxylates and degrades at least HIF-1α [38]. In line with this and the HIFα zonation, HIF prolyl 4-hydroxylases exhibit a zonal distribution with predominant perivenous expression [39]; no zonation pattern has been so far described for FIH.

## 5. HIFs, Redox, and HIF Prolyl 4-Hydroxylases in Liver

Several studies underlined the importance of HIFs for liver integrity. Thereby, HIF-1α and HIF-2α seem to have overlapping but different roles. In particular, hepatocyte-specific absence of HIF-1α showed lobule extension, increased mtDNA content, enhanced lobular oxygen consumption [40], and impaired gluconeogenesis during liver regeneration [41]. HIF-2α has its major roles in hepatic insulin signaling [42], fatty acid beta-oxidation, lipogenesis, and lipid storage [43]. The more dominant role of HIF-2α for liver metabolism was further elucidated in mice with hepatocyte-specific loss of the common beta HIF subunit (ARNT); these mice displayed increased fed insulin levels, lipogenesis, and decreased ketone bodies [44]. 

Although all HIF prolyl 4-hydroxylases act on HIFs, they appear to also have certain differences, at least in liver. While liver-specific loss of HIF prolyl 4-hydroxylase-2 (PHD2) induced HIF-1α, lack of PHD3 induced HIF-2α. Apparently, loss of all three enzymes allowed hepatic expression of the HIF-2 target gene erythropoietin, a pattern normally mainly seen during embryonic life [45].

Appearance of reactive oxygen species (ROS) is associated with the use of O_2_ and in line with the periportal to perivenous differences in mitochondria and oxidative capacities [4,46,47]. Further, ROS were shown to be important regulators of HIFs. They can act either directly on HIFs [48], the HIF coactivators steroid receptor coactivator-1 and transcription intermediary factor 2 [49], or indirectly via NADPH oxidase 4 (NOX4) [50], mitochondrial complex III [51], HIF hydroxylases (PHDs/FIH) [52], kinases and phosphatases, as well as the redox-sensitive transcription factors NF-κB [53,54,55,56,57,58] and NRF2 (Nfe2l2 nuclear factor, erythroid 2-like 2) [59,60].

The complexity of the arising and existing cross talks between all the above modifiers is particularly exemplified by the interplay of HIFs with NF-κB and NRF2. Interestingly, the *HIF1a* gene is a target of NF-κB [53,54,55,56,57] and reciprocally NF-κB can be activated by hypoxia [61]. While the NF-κB p65 subunit abundance was higher in the periportal area, the nuclear appearance of NF-κB p65 followed the oxygen gradient and was higher in the perivenous hepatocytes [62] where higher HIF-1α mRNA levels were found [27]. Likewise, NRF2 was suggested to regulate *HIF1A* gene expression [59,60] and indeed a functional NRF2 antioxidant response element at the *HIF1A* gene could be identified [60]. The interplay of HIF-1α with NRF2 in zonation becomes evident from findings of Nrf2-deficient livers. Apart from being reduced in size, the *Nrf2*-lacking livers display an impaired vascularization, a process in which HIFs are key. Further, a congenital vascular shunt connecting the portal vein and vena cava increased the perivenous O_2_ content and reduced Cyp2e1 expression. By contrast, the normally periportal phosphoenolpyruvate carboxykinase was then also expressed in the perivenous zone [63]. 

Together, these findings indicate that the hypoxia-signaling and redox-signaling pathways have an integrated contribution to metabolic zonation.

## 6. HIFs, Beta-Catenin, and Zonation

As mentioned above, there is no doubt that the Wnt/β-catenin pathway is important for metabolic zonation (for review, see [1]). However, a number of studies indicate that hypoxia and Wnt/β-catenin signaling are also interconnected. In hypoxia in mice (10% O_2_ for 6–72 h) [64], HIF-1α and HIF2α in cells were shown to promote β-catenin´s transcriptional activity [65,66]; the most clear-cut evidence came from the genetic ablation of the *Hif-1a* gene or the *Arnt* gene, which reduced Wnt/β-catenin target gene expression under hypoxia [65]. Further, HIF-1α undergoes a physical interaction with β-catenin [67] and is suggested to promote cell survival, especially under hypoxia. However, this regulation, and the secretion of Wnt3a proteins in particular, can be compromised under very severe, almost anoxic conditions (pO_2_ < 0.01%) in a HIF-independent manner [68]. 

In support of the interplay between the HIF and β-catenin pathway for metabolic zonation is the finding that the negative β-catenin regulator APC is a HIF-1α target gene [69]. Vice versa, APC was found to repress HIF-1α [70] by involving mitochondrial ROS production [71,72,73]. Like HIF signaling, β-catenin signaling is known to be modulated by ROS [74], in particular superoxide and H_2_O_2_ [75]. In support, deletion of the superoxide scavenging manganese superoxide dismutase (MnSOD; *sod2*) in hepatocytes disrupted zonal gene expression [76] and reduced HIF-1α as well as β-catenin levels [77]. 

Thus, low perivenous pO_2_ could promote HIF function, which mediates APC repression and, as a consequence, contributes to β-catenin activation. Vice versa, the high pO_2_ and ROS in the periportal zone would induce APC function and suppress β-catenin signaling.

Although this picture may be appealing, there are quite a number of differences and open questions. For example, there appear to be different functions and regulatory levels between APC and β-catenin. This is highlighted by the findings that mice lacking APC in the liver show a perivenous expression profile but become lethal [12], whereas mice with absence of β-catenin in the liver remain alive and show a pronounced periportal pattern in the perivenous zone [78].

## 7. HIFs, Hedgehog, and Zonation

Hedgehog (HH) signaling is especially active in liver-damaging situations such as in nonalcoholic fatty liver disease (NAFLD), cirrhosis, and hepatocellular carcinoma (HCC) [79,80]. Accordingly, HH signaling is most active in hepatic stellate cells and cholangiocytes [81,82]; it was also shown to contribute to metabolic zonation in hepatocytes [16]. Three HH proteins (Sonic-HH, Indian-HH, and Desert-HH) are known. The membrane protein Dispatched (DISP) promotes their secretion, which enables their autocrine or paracrine action on receptors called Patched (PTCH1, -2). PTCH has a coreceptor called Smoothened (SMO). SMO in turn regulates nuclear import and activity of the glioma-associated oncogenic transcription factors GLI1, GLI2, and GLI3 [83]. In the absence of HH, PTCH inhibits SMO, thereby preventing nuclear import of GLIs. Once HH binds to PTCH, the inhibitory action of PTCH on SMO is abolished, and as a result, GLIs become transported into the nucleus [83]. 

IHH shows a perivenous zonation in mouse liver [8] and deletion of SMO in hepatocytes lead to lipogenesis mainly via GLI3-mediated upregulation of SREBP1, and enzymes such as the normally perivenous FASN [84,85] in periportal hepatocytes [86]. Other metabolic pathways such as cholesterol biosynthesis, glycolysis, and glycogen storage were not altered, but regulation of periportal IGF1 and perivenous IGFBP1 [87] was reciprocally affected; IGF1 was decreased and IGFBP was increased upon SMO deletion [88]. 

The action of the HH pathway can also be linked to hypoxia signaling, although the details are far from being understood and the results vary from cell-type to cell-type. Hypoxia was able to induce SHH, and PTCH1 expression as well as a systemic HH response in mice via HIF-1α [89]. Apparently, HH response towards hypoxia can also involve HIF-2α, depending on the cell type [90]. Interestingly and likely as a balancing act, hypoxia could also upregulate SMO transcription in different cell models [91,92]. Similar to HH signaling, hypoxia enhanced perivenous FASN expression, not via HIFs but via SREBP1 [93]. Vice versa, hypoxia and HIF-1α inhibited expression of key genes [94] regulating β-oxidation, which is found periportally. Moreover, perivenous oxygen tensions were able to enhance IGFBP-1 expression in a HIF proline hydroxylase- and HIFα-dependent manner [35]. 

**Figure 2:**
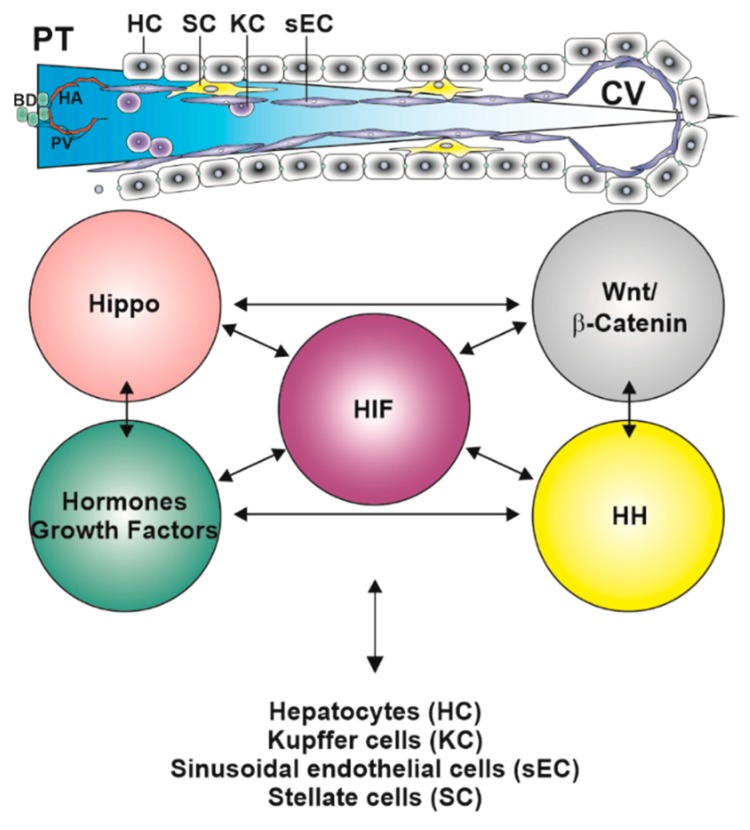
Interplay of major zonation signaling pathways. The sinusoids facilitate free exchange of nutrients, metabolites, and various substrates, such as oxygen. Although the gradient may vary, the periportal to perivenous oxygen gradient (in blue) exists inevitably under almost all conditions. Hypoxia-inducible factors (HIFs) may be major orchestrators since they adapt the gene expression profile in response to changing oxygen tensions. The HIF system in hepatocytes but also in the other sinusoidal cells can undergo cross-talks with other major zonation regulating pathways such as the WNT/β-catenin pathway and associated components, hormone and growth factor signaling pathways, the Hippo pathway and the hedgehog (HH) pathway. All these pathways and factors exempt the oxygen gradient; depend, to the more or lesser extent, on the presence of specific ligands which are secreted from other cells than the hepatocytes, e.g., liver endothelial cells which contribute to the synthesis of certain WNTs, whereas cholangiocytes and stellate cells can secrete hedgehog ligands. PT, portal tract consisting of branches from the hepatic artery (HA), portal vein (PV) and a bile duct; CV, central vein.

Apart from hypoxia signaling, the HH pathway is also at the cross roads with the Hippo and Wnt/β-catenin pathways, which may have a confounding role in shaping zonation and further research would boost our knowledge in this field (Figure 2).

## 8. HIFs, Hippo, and Zonation

Hippo signaling (Salvador–Warts–Hippo pathway) has been identified as a general regulator of organ size and is associated with developmental processes, regeneration, and carcinogenesis not only in liver. In liver, activation of this pathway has been attributed to liver overgrowth (for review see [15]. Similar to β-catenin, canonical and noncanonical signaling aspects are distinguished. In the canonical part, two protein kinases known as serine/threonine-protein kinase-3 and -4 (STK3/STK4) together with the regulator protein salvador family WW domain-containing protein (SAV1) phosphorylates and activates large tumor suppressor kinase-1/-2 (LATS1/2) in complex with its regulatory protein MOB kinase activator-1 (MOB1). LATS-1/2 phosphorylates and inactivates the transcriptional coactivators yes-associated protein 1 (YAP) and WW domain-containing transcription regulator 1 (WWTR1/TAZ). Phosphorylation of YAP inhibits its translocation into the nucleus and promotes its proteasomal degradation (for review see [15]. When the kinase relay is off, nuclear YAP binds to transcriptional enhancer-associated domain (TEAD) transcription factors and promotes expression of genes regulating cell proliferation, cell migration, and survival. As YAP is present in all cell types of the liver and can be modulated by several, including oncogenic, pathways, it can besides proliferation also promote transdifferentiation of hepatocytes towards a biliary or progenitor phenotype [15,95,96,97].

The periportal zone shows highest abundance of nuclear YAP, with biliary cells possessing the highest levels [98]. Since the YAP gradient is reciprocal to that of active β-catenin, it would be tempting to speculate that the Hippo pathway could act as inhibitor of WNT signaling to regulate zonation. In support are findings showing that the perivenous zone expressing glutamine synthase was enlarged upon knockout of YAP and with lack of MST1 and MST2 decreased. In addition, nuclear β-catenin levels were reduced in mice lacking MST1 and MST2 [96]. However, both activation of the WNT pathway via R-spondin and overexpression of YAP can induce liver growth [95,99], hence arguing against an antagonistic role of WNT and Hippo for zonation. Thus, more studies are needed to unravel the interaction of these two pathways for liver zonation.

Hypoxia and HIFs affect Hippo pathway components at different levels. Although not in liver, HIF-1α, but not HIF-2α was shown to be a direct inducer of TAZ expression [100]. In addition, HIF-1α was able to increase expression of the LATS2-degrading ubiquitin ligase SIAH1; consequently TAZ´ nuclear translocation becomes promoted [100]. Further, TAZ was also shown to interact with HIF-1α in metastasizing breast cancer cells [101], thereby stimulating TAZ/TEAD transcriptional activity. Vice versa, TAZ augmented HIF-1α transactivity and expression of its target genes [102]. Furthermore, hypoxia promoted YAP binding to HIF-1α in the nucleus of the HepG2 and Huh7 cells, which then displayed accelerated glycolysis [103]. 

Considering the relationship between HIF and Hippo pathways, it is of note that, again, the intercellular interplay has to be considered. Apparently, YAP activation in sinusoidal endothelial cells has been shown to promote angiogenesis in fibrotic livers via the HIF-1α and VEGF-A axis [104,105]. Moreover, YAP, particularly in hepatocytes and hepatic stellate cells of regenerating liver, was able to activate HH signaling [106] and needs to be seen in connection to other pathways such as Hippo with Notch [107], and Notch with HIF [108].

## 9. HIFs, Steatosis, and Fatty Liver Disease

### 9.1. HIFs and NAFLD

Fatty liver disease is commonly divided into nonalcoholic fatty liver disease (NAFLD) and alcoholic fatty liver disease (ALD). Steatosis, i.e., the appearance of lipid droplets in more than 5% of hepatocytes [109] is one characteristic aspect of them. NAFLD´s prevalence worldwide is about ~24% [110], and the lipids commonly arise from overnutrition; consequently, NAFLD is associated with the pandemic occurrence of obesity, type-2 diabetes (T2D), and cardiovascular disease [111,112]. NAFLD can manifest as benign perivenous steatosis without inflammation or can be accompanied by ballooning and inflammation, a situation known as nonalcoholic steatohepatitis (NASH). About 10–30% of NASH patients show progression into fibrosis and will develop cirrhosis within ten years after diagnosis [113]. From the patients diagnosed with cirrhosis, about 40–60% will develop hepatocellular carcinoma (HCC) [114,115]. 

Although the two-hit hypothesis describes several modular aspects driving the development from steatosis to NASH, the detailed underlying mechanisms are largely unknown. Apart from the excess of nutrients and insulin resistance, ER stress, impaired autophagy, and reactive oxygen species (ROS)-mediated lipid oxidation [116,117], recent data and mathematical modeling indicate that hypoxia may promote disease progression [9,118,119,120]. Indeed, hepatocellular injury in NASH is mainly found in the perivenous zone in line with the perivenous zonation of fatty acid synthesis [121]. Further, the marked zonation of several molecular species of phosphatidylcholine [122] present in controls and patients with simple steatosis is lost in NASH patients [123]. The participation of the HIF system was further supported by the appearance of steatosis in mice with enhanced HIF expression due to lack of the HIF destruction protein VHL. With respect to HIFs, HIF-2α seems to be more powerful than HIF-1α with respect to lipid storage [124] and insulin sensitivity [42] and to share only some target genes with HIF-1α [124]. In particular, constitutive expression of HIF-2α, but not HIF-1α, in hepatocytes enhanced expression of lipogenic genes such as SREBP1c, FASN and promoted lipid storage as well as steatosis. At the opposite, β-oxidation regulators such as PPARα, and carnitine palmitoyl-CoA transferase-1 were decreased [43]. In addition, HIF-2α was shown to be the major pexophagy driver [125]. Peroxisomes are a major site of fatty acid degradation and under normal circumstances, they consume ~20% of O_2_ and produce ~35% of H_2_O_2_ in liver [126]. Thus, a reduction in their number would decrease their metabolic activity and O_2_ consumption under hypoxia. Indeed, the HIF-2α-driven pexophagy and PPARα antagonism [125,127] changed the lipid profile in mice to a NAFLD-like pattern [128]. In line with this are findings from mice, where a choline-deficient L-amino acid-deficient- or a methionine/choline-deficient diet-dependent NAFLD was ameliorated due to hepatocyte-specific deletion of HIF-2α and by concomitant decreased macrophage M1 polarizing histidine-rich glycoprotein (HRGP) expression [129], which links HIF-2α to inflammation and NASH. A further, causative role for HIF-2α in NAFLD came from a recent study with obesity-induced hypoxia in intestinal epithelial cells where the HIF-2α-specific inhibitor PT2385 was of benefit against metabolic diseases [130].

While being less powerful in lipid metabolism, HIF-1α has a stronger effect on carbohydrate metabolism, mitochondrial function, and structural maintenance [40,41]. In addition to the gene products involved in glucose uptake and glycolysis [26,131], it enhances pyruvate dehydrogenase kinase-1, -3, and -4 expression and thus blocks pyruvate entry into mitochondria [132,133]. HIF-1α is also a reprogrammer of the mitochondrial electron transport chain function. It induces the electron transport complex I protein NADH dehydrogenase (ubiquinone) 1 alpha subcomplex, 4-like 2 (NDUFA4L2) [134]. Further, in complex IV, it promotes exchange of cytochrome c oxidase COX4-1 with COX4-2, which leads to reduced oxygen consumption and decreases ROS [135]. HIF-1α also contributes to selective autophagy; it promotes mitophagy and blocks Fe/S cluster assembly by inducing BNIP3 [136] and miRNA-210 [137]. Together, hypoxia and HIFs, HIF-2α in particular, have a role in NAFLD.

### 9.2. HIFs and ALD

Ethanol, commonly called alcohol, is consumed at a rate of 20–30 g daily in many Western cultures and represents a major cause of chronic liver disease. Importantly, obesity constitutes a high-risk progressive factor for ALD [138], although other diseases, e.g., hepatitis B or C virus infections, and ethnic as well as gender differences cannot be neglected [139,140]. Similar to NAFLD, ALD displays features ranging from steatosis, hepatitis, fibrosis, to cirrhosis and HCC. The prevalence of steatosis and cirrhosis in heavy drinkers is ~90% and 30%, respectively, with the latter being present in ~50% of all death cases caused by liver failure [141,142]. About 10% of cirrhosis patients will develop HCC [139].

The pathogenesis of ALD is complex and not understood to the last detail. One arm in ALD development consists of an ethanol-modulated intestinal barrier and gut microbiome where Gram-negative bacteria become enriched. Leakage of bacterial products from the gut into the circulation activates Kupffer cells as part of the immune system; consequently, they release cytokines such as TNFα as well as ROS. The second arm is supposed to induce oxygen consumption, oxidative stress, loss of protection, and apoptosis directly on hepatocytes [143,144,145,146]. Consequently, this causes hypoxia as seen in animal models and ALD patients, which is aggravated in the perivenous zone [147,148]. 

The role of HIFs in ALD is not fully understood. As they contribute to homeostasis under normal conditions, they could either contribute to the apoptotic process or to tissue protection. Currently, only reports on HIF-1α are available and they do not allow an entirely concise view. One study reported that hepatocyte-specific HIF-1α deletion in mice was protective against an ethanol containing Lieber-DeCarli diet [149], whereas another study with the same animal model and almost identical dietary protocol found that depletion of HIF-1α in hepatocytes aggravated steatosis [150]. Mechanistically, the latter involved induction of SREBP-1c and its targets FASN and acyl-CoA carboxylase (ACC)-1 due to derepression of SREBP-1c by HIF-target differentiated embryo chondrocyte 1 (DEC1) [150]. 

The latter and earlier findings on liver regeneration [40] imply that HIFs, particularly HIF-1α, promote a tissue-protective program. Indeed, a recent study from hypomorphic mice with systemic inactivation of the main HIF prolyl hydroxylase-2 (PHD2/EGLN1) showed less adiposity, an improved lipoprotein profile, and insulin sensitivity than the wild-type mice upon feeding an ethanol diet [151]. It appeared that the protective effects were mediated by antioxidant HIF target genes, such as the monocarboxylate transporters solute carrier family 16 members 1 and 3 and the GSH synthesizing glutathione cysteine ligase, as well as a higher catalytic activity of aldehyde dehydrogenase 2. Similarly, pharmacological HIF prolyl hydroxylase-2 inhibition improved handling of the toxic ethanol metabolites and oxidative stress [151].

## 10. HIFs, Liver Fibrosis, Cirrhosis, and HCC

The progression to fibrosis, cirrhosis, and HCC from NAFLD or ALD is a complex process and involving hepatocytes, Kupffer-, stellate-, and endothelial cells. In particular, the structural changes in sinusoidal architecture affect blood flow causing hypoxia [152], as visualized by HIF-target gene expression in human liver samples [153] and mouse models of liver fibrosis [154,155]. While the role of HIF-2α in fibrosis is still unknown, deletion of HIF-1α in cells of the myeloid lineage reduced bile duct ligation-induced fibrosis in mice [156]. In addition, HIF-1α was shown to drive angiogenesis and collagen expression in stellate cells [157]. Further, the chemokine receptor CXCR4 and its ligand stromal cell-derived factor-1 (SDF-1) are HIF-target genes [158] and both promote infiltration of inflammatory cells. 

The fibrotic process is accompanied by epithelial–mesenchymal transition (EMT). During EMT, E-cadherin expression is decreased and, vice versa, α-smooth muscle actin and vimentin expression are increased. E-cadherin expression is restricted to periportal hepatocytes, whereas N-cadherin is enriched in perivenous hepatocytes and NASH disrupts this zonal expression of cadherins [159]. Both HIF-1α and HIF-2α can regulate EMT by inducing the E-cadherin repressors SNAIL1, TWIST1, transcription factor 3, and the Zinc Finger E-Box Binding Homeobox 1 and 2 [160,161,162,163,164]. While these mechanisms seem to promote EMT, fibrosis, and eventually HCC, they also represent a feedback in NAFLD-associated insulin resistance. Indeed, hepatocyte-specific SNAIL1 overexpression reduced insulin-stimulated lipogenesis by epigenetically repressing FASN promoter activity. By contrast, hepatocyte-specific SNAIL1 deletion aggravated NAFLD [165]. Thus, these findings imply that the HIF system may have a dual role: While being rather lipogenic under normal conditions, the challenge of the system under obesity conditions could, in part via SNAIL1 and epigenetic mechanisms counteract NAFLD. However, further data are needed to prove this hypothesis. 

From a worldwide perspective, HCC is the second leading cause of cancer-related deaths, with a median overall survival between six to eight months. Males are twice as affected than women [166,167]. The pathogenesis of HCC is based on chronic disease. While NAFLD/NASH, ALD, drug abuse or congenital disorders account only for ~20% of the HCC cases, chronic hepatitis B and C infections account for ~80% of the HCC cases [168]. The detection of specific HIF1a gene variants suitable to predict HCC susceptibility [169,170] implied participation of the HIF system in this process. Indeed, higher HIF-1α and HIF-2α levels were detected in HCC [171,172] and particularly HIF-1α showed a positive correlation with HCC grade, and metastasis, but was negatively correlated with the overall survival rate [171,172,173,174,175,176,177]. Further, the hepatitis virus B X-protein showed a positive correlation with HCC and induced levels of HIF-1α in cell cultures [173]. In addition, HCV glycoproteins upregulated SNAIL and TWIST expression in a HIF-1 α-dependent manner and promoted EMT [178]. The role of HIF-2α in HCC is less clear and it was found to either promote [172,176] or to inhibit it [179].

Interestingly, the HCC drugs sorafenib, regorafenib, lenvatinib, cabozantinib, and ramucirumab, being either used or in phase III clinical trials, act against processes of growth factors, which are virtually all directly or indirectly regulated by the HIF system [26,180]. Together, these findings suggest participation of HIF-1α in HCC pathogenesis, while the exact role of HIF-2α needs further resolution. 

The role of the HIF system for HCC pathogenesis is further underlined by mouse studies where HIF-prolyl hydroxylase-2 (PHD2/EGLN1) heterozygous mice displayed more HCC growth and presence of cholangiocarcinoma in response to diethylnitrosamine. These effects appeared to involve cross talk with the NOTCH pathway at a late stage of nodule formation.

Although conflicting data with respect to HIF-prolyl hydroxylase-2 (PHD2/EGLN1) in patient HCC were reported [181,182], HIF-prolyl hydroxylase-3 (PHD3/EGLN3) acts as a favorable marker [183,184]. Although the current findings about the HIF system in HCC suggest that HIFs would not be beneficial, one would also need to take cell-type specific effects as well as the interaction with the immune system into account. Given the impaired vessel architecture in a specific phase of fibrosis, cirrhosis, or HCC it may well be that HIF would promote vessel regeneration and blood supply. Indeed, findings from heterozygous HIF-prolyl hydroxylase-2 ^+/−^ (PHD2/EGLN1^+/−^) mice support this by showing an improved vessel architecture within the tumor but reduced metastasis upon transplantation of PHD2/EGLN1^+/+^ tumor cells compared to wild-type mice [185]. 

Considering this, HIF-prolyl hydroxylase inhibitors could be useful in HCC. Preclinical studies in mice showed that treatment with the HIF-prolyl hydroxylase pan-inhibitor ethyl-3,4-dihydroxybenzoate augmented liver regeneration after partial hepatectomy or portal vein ligation and did not promote growth of colorectal cancer cell metastases in the liver [186]. In humans, only roxadustat was examined in an open-label study with eight persons with normal hepatic function and eight patients with liver cirrhosis (Child–Pugh score 7–9). A single oral dose of 100 mg roxadustat in fasting patients did not show clinically significant differences, despite a moderate impairment on the pharmacokinetics and pharmacodynamics, indicating that these drugs were well tolerated and did not aggravate the cirrhosis within the investigated time frame of 96 h [187]. Together, the therapeutic potential of HIF-prolyl hydroxylase inhibitors in human liver diseases awaits further investigation.

## 11. HIFs in Ischemia-Reperfusion-Mediated Liver Injury

Ischemia-reperfusion (I/R)-mediated liver injury significantly contributes to morbidity and mortality in patients who have undergone liver surgery, in particular transplantation [188]. I/R occurs primarily during liver surgery including resection of large intrahepatic lesions and organ preservation prior to transplantation. During the ischemic period, the lack of oxygen and nutrients causes an imbalance in ATP supply and demand, resulting in a number of cellular processes, including protective functions, not being fully executed. Upon restoration of the blood flow, the liver is subjected to a further insult, where ROS-mediated protein and lipid oxidation are thought to aggravate the injury causing tissue damage and inflammatory responses. Overall, this causes a poor, if not dysfunction, of the graft [189]. 

Although the cellular and molecular mechanisms accounting for I/R liver injury are not fully resolved, it appears that ischemic preconditioning and activation of HIFs are important in protecting the liver from I/R injury. Indeed, ischemic preconditioning by clamping the hepatic artery has been shown to stabilize HIF-1α and to promote cell survival during I/R liver injury [190,191], similar to cardiac protection during I/R injury. This was further supported by pharmacological studies where drugs such as losartan, mangafodipir, or ethyl 3,4-dihyroxybenzoate caused, among protection against I/R injury, stabilization of at least HIF-1α; no data on the other HIFs are available from these studies [190,192,193,194]. The protective effects were then mediated via HIF-dependent expression of genes that regulate multiple pathways, such as cellular energy metabolism, angiogenesis, and well-described protective molecules, including heat-shock proteins, heme oxygenase-1, nitric oxide, and adenosine [190,192,195,196]. 

More specifically, it was then shown that lack of the HIF-prolyl hydroxylase-1 gene (Phd1/Egln2) in mice protects against acute I/R liver injury [195]. Mechanistically, this involves reduction of oxidative stress, HIF-mediated reprogramming of hepatocellular metabolism [195], and activation of NF-κB signaling [197]. For the latter, proline 191 in the beta subunit of the I-kappaB kinase appears to be a crucial HIF-prolyl hydroxylase-1 substrate [197]. In line with the reduced apoptosis, HIF-prolyl hydroxylase-1-lacking livers showed a higher potential for liver regeneration that was linked to enhanced activity of cMYC [198]. Moreover, the protective effect of HIF-prolyl hydroxylase-1 depletion appears not to be limited to the liver. Experiments with HIF-prolyl hydroxylase-1 deficient mice showed that the lack of HIF-prolyl hydroxylase-1 attenuates myocardial I/R injury, likely by inducing signaling cross-talk between HIF-1α, β-catenin, endothelial nitric oxide synthase, NF-kB, and Bcl-2 [199]. Likewise, data from HIF-prolyl hydroxylase-2 hypomorphic mice show that HIF-prolyl hydroxylase-2 deficiency protects against I/R injury in heart and skeletal muscle [200,201]. In line, depletion of HIF-prolyl hydroxylase-3 also protected hearts from I/R injury, which was accompanied by increased HIF-1α stabilization and an inhibition of the DNA damage response at the level of CHK1 and p53 [202]. 

While the data with respect to pharmacological and genetic prolyl hydroxylase inhibition are not restricted to any particular HIF transcription factor, the proper role of each HIFα subunit may vary with respect to organ or cells being involved. Although no data on HIF-1α and HIF-2α in acute I/R liver injury are available yet, experiments in heterozygous HIF-1α mice (Hif1a^+/−^) showed that HIF-1α-mediated IL10 expression is involved in protection of the heart by remote ischemic preconditioning [203]. Further, mice in which HIF-1α was deleted in Tie2(+) bone marrow and vascular endothelial cells showed a complete absence against ischemic preconditioning in the heart [204]. By contrast, deletion of HIF-1α or HIF-2α in endothelial cells of mice with an ischemic kidney injury model showed that endothelial HIF-2α, but not endothelial HIF-1α, promoted recovery from ischemic kidney injury. In addition, pharmacological inhibition of HIF prolyl-hydroxylases with GSK1002083A as well as genetic inactivation of endothelial HIF-prolyl hydroxylase-2 protected mice from ischemic kidney injury [205]. Similarly, a recent study in mice with inducible deletion of HIF-1α or HIF-2α in cardiac myocytes showed that loss of HIF-2α increases infarct sizes and that HIF-2α-dependent upregulation of the growth factor amphiregulin (AREG) mediates cardioprotection [206]. Thus, the animal experiments in different ischemia injury models suggest a cell-type selective involvement of the two HIF transcription factors with a so-far unexpected role of HIF-2α. Whether similar phenomena with respect to HIF-1α or HIF-2α in the different cell types of the liver such as liver endothelial cells, stellate cells, Kupffer cells, pit cells or hepatocytes will be seen remains to be investigated. Although all these studies support the connection of the HIF-prolyl hydroxylase and HIFs in I/R injury, a recent study reported that systemic or skeletal muscle-specific HIF-prolyl hydroxylase-2 inactivation can also protect mice against myocardial I/R injury in a HIF-independent manner by hepatic production and secretion of kynurenic acid [207]. 

Collectively, these data indicate that both inactivation of HIF-prolyl hydroxylases and/or activation of the HIF-response can exert protective effects against I/R injury in several organs and not only in liver. However, more knowledge about involvement of the specific HIFα subunits in selective cell-types and the detailed contribution of HIF-independent HIF-prolyl hydroxylase inhibitory effects would broaden the therapeutic opportunities in I/R injury and are therefore urgently needed, especially in view of their beneficial effects for liver transplantation.

## 12. Hypoxia, HIF, and HIF-Prolyl Hydroxylase-Related Therapies to Treat Liver Diseases

**Figure 3:**
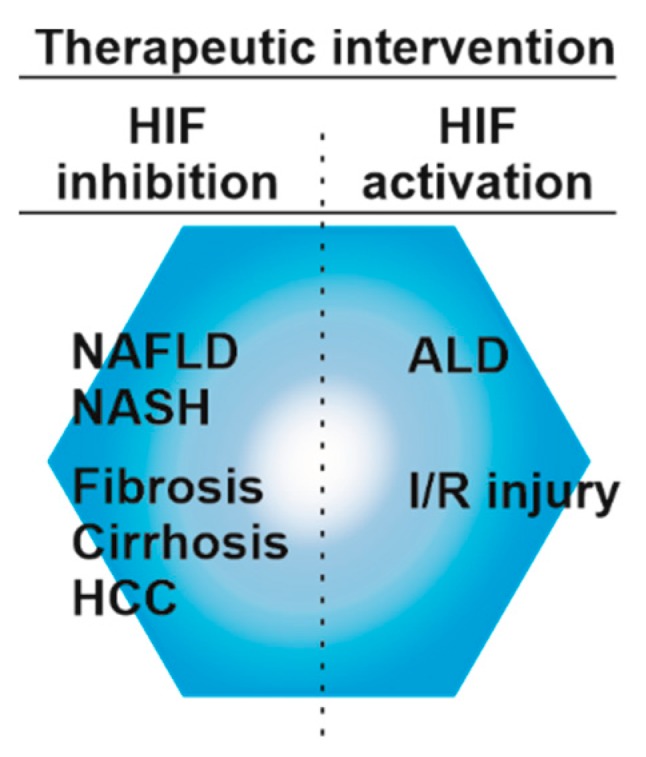
Targeting HIF-signaling for the treatment of liver diseases. Activation of HIF signaling appears to be beneficial during ischemia-reperfusion (I/R) injury and partially in ALD. Activation of HIFs can be achieved by HIF prolyl hydroxylase inhibitors. In NAFLD, NASH fibrosis, cirrhosis, and HCC inhibition of HIFs may be of benefit.

As orally administrable HIF-prolyl hydroxylase inhibitors have been approved for the treatment of anemia in chronic kidney disease patients on dialysis in China and are awaiting approval in other countries [208], the possible use of these inhibitors for certain aspects of liver diseases could be envisioned (Figure 3). 

Apparently, the majority of studies support the view that HIF-prolyl hydroxylase inhibitors might be of benefit in acute liver injury, such as drug-induced or I/R injury. Hence, it is a scenario where HIF-prolyl hydroxylase inhibitors could be administered to organ donors before surgery or after to promote liver regeneration in alive donors; alternatively, they could be simply added into the perfusate of the explant. 

HIF-prolyl hydroxylase inhibition in chronic liver diseases such as NAFLD/NASH, fibrosis, cirrhosis and HCC is, based on current data, not of benefit. This is even more complicated by the multiple cross-talks of the HIF system with various pathways, particularly Wnt, Hedghog, Hippo, and NF-κB in liver. However, there might be, dependent on the dose, time, and features such as selectivity, targeted cell type, and/or HIF variant, a potential use of either inhibitors or activators of the HIF system. Considering this and the poor prognosis, high risks of HCC recurrence, metastases, and limited treatment options [168,209], it is necessary and of therapeutic value to gain more insight into the role of the HIF system during HCC pathogenesis.

## 13. Conclusions

With the increase in the prevalence of liver diseases and general metabolic diseases that perturb liver functions and are accompanied by malfunction of systemic circulation, fundamental aspects addressing the oxygen content and the role of HIFs on metabolic liver zonation and related diseases such as fatty liver disease, fibrosis, or hepatocellular carcinoma are emerging. A number of observations from different model systems indicate that hypoxia, HIF-prolyl hydroxylases, and HIFs are linked with various aspects of liver zonation and the pathogenesis of acute or chronic liver diseases. However, future studies are necessary to define the role of the HIF system in the context of cell–cell interactions and how it integrates various and competing growth signals for liver homeostasis and zonation maintenance. Although recent technical advantages allow creating combined cell-by-cell RNA sequence maps of liver lobules [7] and fate-mapping [97] of hepatocytes responding to different liver injuries, more research is needed to fully define the signals regulating the interplay of the various signaling pathways contributing to zonation. Moreover, further studies are required to understand how oxygen-dependent and -independent activation/inactivation of the HIF pathway acts in context of various chronic liver injuries.

## References

[1] Kietzmann T. (2017). Metabolic zonation of the liver: The oxygen gradient revisited. Redox Biol..

[2] Sasse D., Spornitz U.M., Maly I.P. (1992). Liver architecture. Enzyme.

[3] Kietzmann T., Dimova E.Y., Flügel D., Scharf J.G. (2006). Oxygen: Modulator of physiological and pathophysiological processes in the liver. Z. Gastroenterol..

[4] Novikoff A.B. (1959). Cell heterogeneity within the hepatic lobule of the rat: Staining reactions. J. Histochem. Cytochem..

[5] Loud A.V. (1968). A quantitative stereological description of the ultrastructure of normal rat liver parenchymal cells. J. Cell Biol..

[6] Jungermann K., Sasse D. (1978). Heterogeneity of liver parenchymal cells. Trends Biochem. Sci..

[7] Halpern K.B., Shenhav R., Matcovitch-Natan O., Toth B., Lemze D., Golan M., Massasa E.E., Baydatch S., Landen S., Moor A.E. (2017). Single-cell spatial reconstruction reveals global division of labour in the mammalian liver. Nature.

[8] Gebhardt R., Matz-Soja M. (2014). Liver zonation: Novel aspects of its regulation and its impact on homeostasis. World J. Gastroenterol..

[9] Schleicher J., Tokarski C., Marbach E., Matz-Soja M., Zellmer S., Gebhardt R., Schuster S. (2015). Zonation of hepatic fatty acid metabolism—The diversity of its regulation and the benefit of modeling. Biochim. Biophys. Acta.

[10] Jungermann K., Kietzmann T. (1996). Zonation of parenchymal and nonparenchymal metabolism in liver. Annu. Rev. Nutr..

[11] Halpern K.B., Shenhav R., Massalha H., Toth B., Egozi A., Massasa E.E., Medgalia C., David E., Giladi A., Moor A.E. (2018). Paired-cell sequencing enables spatial gene expression mapping of liver endothelial cells. Nat. Biotechnol..

[12] Benhamouche S., Decaens T., Godard C., Chambrey R., Rickman D.S., Moinard C., Vasseur-Cognet M., Kuo C.J., Kahn A., Perret C. (2006). APC tumor suppressor gene is the “zonation-keeper” of mouse liver. Dev. Cell.

[13] Planas-Paz L., Orsini V., Boulter L., Calabrese D., Pikiolek M., Nigsch F., Xie Y., Roma G., Donovan A., Mart P. (2016). The RSPO-LGR4/5-ZNRF3/RNF43 module controls liver zonation and size. Nat. Cell Biol..

[14] Braeuning A., Menzel M., Kleinschnitz E.-M., Harada N., Tamai Y., Köhle C., Buchmann A., Schwarz M. (2007). Serum components and activated Ha-ras antagonize expression of perivenous marker genes stimulated by β-catenin signaling in mouse hepatocytes. FEBS J..

[15] Patel S.H., Camargo F.D., Yimlamai D. (2017). Hippo Signaling in the Liver Regulates Organ Size, Cell Fate, and Carcinogenesis. Gastroenterology.

[16] Matz-Soja M., Hovhannisyan A., Gebhardt R. (2013). Hedgehog signalling pathway in adult liver: A major new player in hepatocyte metabolism and zonation?. Med. Hypotheses.

[17] Cheng X., Kim S.Y., Okamoto H., Xin Y., Yancopoulos G.D., Murphy A.J., Gromada J. (2018). Glucagon contributes to liver zonation. Proc. Natl. Acad. Sci. USA.

[18] Stanulovic V.S., Kyrmizi I., Kruithof-de Julio M., Hoogenkamp M., Vermeulen J.L., Ruijter J.M., Talianidis I., Hakvoort T.B., Lamers W.H. (2007). Hepatic HNF4α deficiency induces periportal expression of glutamine synthetase and other pericentral enzymes. Hepatology.

[19] Sekine S., Ogawa R., Mcmanus M.T., Kanai Y., Hebrok M. (2009). Dicer is required for proper liver zonation. J. Pathol..

[20] Russell J.O., Monga S.P. (2018). Wnt/β-Catenin Signaling in Liver Development, Homeostasis, and Pathobiology. Annu. Rev. Pathol..

[21] Blitzer J.T., Nusse R. (2006). A critical role for endocytosis in Wnt signaling. BMC Cell Biol..

[22] Rocha A.S., Vidal V., Mertz M., Kendall T.J., Charlet A., Okamoto H., Schedl A. (2015). The Angiocrine Factor Rspondin3 Is a Key Determinant of Liver Zonation. Cell Rep..

[23] Choi S.S., Omenetti A., Syn W.K., Diehl A.M. (2011). The role of Hedgehog signaling in fibrogenic liver repair. Int. J. Biochem. Cell Biol..

[24] Leibing T., Geraud C., Augustin I., Boutros M., Augustin H.G., Okun J.G., Langhans C.D., Zierow J., Wohlfeil S.A., Olsavszky V. (2018). Angiocrine Wnt signaling controls liver growth and metabolic maturation in mice. Hepatology.

[25] Prabhakar N.R., Semenza G.L. (2012). Adaptive and maladaptive cardiorespiratory responses to continuous and intermittent hypoxia mediated by hypoxia-inducible factors 1 and 2. Physiol. Rev..

[26] Semenza G.L. (2014). Oxygen sensing, hypoxia-inducible factors, and disease pathophysiology. Annu. Rev. Pathol..

[27] Kietzmann T., Cornesse Y., Brechtel K., Modaressi S., Jungermann K. (2001). Perivenous expression of the mRNA of the three hypoxia-inducible factor α-subunits, HIF1α, HIF2α and HIF3α, in rat liver. Biochem. J..

[28] Wenger R.H., Stiehl D.P., Camenisch G. (2005). Integration of oxygen signaling at the consensus HRE. Sci. STKE.

[29] Smythies J.A., Sun M., Masson N., Salama R., Simpson P.D., Murray E., Neumann V., Cockman M.E., Choudhry H., Ratcliffe P.J. (2019). Inherent DNA-binding specificities of the HIF-1α and HIF-2α transcription factors in chromatin. EMBO Rep..

[30] Löfstedt T., Fredlund E., Holmquist-Mengelbier L., Pietras A., Ovenberger M., Poellinger L., Påhlman S. (2007). Hypoxia inducible factor-2a in cancer. Cell Cycle.

[31] Heikkila M., Pasanen A., Kivirikko K.I., Myllyharju J. (2011). Roles of the human hypoxia-inducible factor (HIF)-3α variants in the hypoxia response. Cell. Mol. Life Sci..

[32] Hara S., Hamada J., Kobayashi C., Kondo Y., Imura N. (2001). Expression and characterization of hypoxia-inducible factor (HIF)-3α in human kidney: Suppression of HIF-mediated gene expression by HIF-3α. Biochem. Biophys. Res. Commun..

[33] Makino Y., Kanopka A., Wilson W.J., Tanaka H., Poellinger L. (2002). Inhibitory PAS domain protein (IPAS) is a hypoxia-inducible splicing variant of the hypoxia-inducible factor-3α locus. J. Biol. Chem..

[34] Zhang P., Lu L., Yao Q., Li Y., Zhou J., Liu Y., Duan C. (2012). Molecular, functional, and gene expression analysis of zebrafish hypoxia-inducible factor-3α. Am. J. Physiol. Regul. Integr. Comp. Physiol..

[35] Scharf J.G., Unterman T.G., Kietzmann T. (2005). Oxygen-dependent modulation of insulin-like growth factor binding protein biosynthesis in primary cultures of rat hepatocytes. Endocrinology.

[36] Kaelin W.G., Ratcliffe P.J., Semenza G.L. (2016). Pathways for oxygen regulation and homeostasis: The 2016 albert lasker basic medical research award. JAMA.

[37] Koivunen P., Kietzmann T. (2018). Hypoxia-Inducible Factor Prolyl 4-Hydroxylases and Metabolism. Trends Mol. Med..

[38] Ginouvès A., Ilc K., Macías N., Pouysségur J., Berra E. (2008). PHDs overactivation during chronic hypoxia “desensitizes” HIFa and protects cells from necrosis. Proc. Natl. Acad. Sci. USA.

[39] Khan Z., Michalopoulos G.K., Stolz D.B. (2006). Peroxisomal localization of hypoxia-inducible factors and hypoxia-inducible factor regulatory hydroxylases in primary rat hepatocytes exposed to hypoxia-reoxygenation. Am J. Pathol..

[40] Tsukada K., Tajima T., Hori S., Matsuura T., Johnson R.S., Goda N., Suematsu M. (2013). Hypoxia-inducible factor-1 is a determinant of lobular structure and oxygen consumption in the liver. Microcirculation.

[41] Tajima T., Goda N., Fujiki N., Hishiki T., Nishiyama Y., Senoo-Matsuda N., Shimazu M., Soga T., Yoshimura Y., Johnson R.S. (2009). HIF-1a is necessary to support gluconeogenesis during liver regeneration. Biochem. Biophys. Res. Commun..

[42] Wei K., Piecewicz S.M., McGinnis L.M., Taniguchi C.M., Wiegand S.J., Anderson K., Chan C.W., Mulligan K.X., Kuo D., Yuan J. (2013). A liver Hif-2α-Irs2 pathway sensitizes hepatic insulin signaling and is modulated by Vegf inhibition. Nat. Med..

[43] Rankin E.B., Rha J., Selak M.A., Unger T.L., Keith B., Liu Q., Haase V.H. (2009). Hypoxia-inducible factor 2 regulates hepatic lipid metabolism. Mol. Cell. Biol..

[44] Wang X.L., Suzuki R., Lee K., Tran T., Gunton J.E., Saha A.K., Patti M.-E., Goldfine A., Ruderman N.B., Gonzalez F.J. (2009). Ablation of ARNT/HIF1ß in Liver Alters Gluconeogenesis, Lipogenic Gene Expression, and Serum Ketones. Cell Metab..

[45] Minamishima Y.A., Kaelin W.G. (2010). Reactivation of hepatic EPO synthesis in mice after PHD loss. Science.

[46] Schmucker D.L., Mooney J.S., Jones A.L. (1978). Stereological analysis of hepatic fine structure in the Fischer 344 rat. Influence of sublobular location and animal age. J. Cell Biol..

[47] Jungermann K. (1988). Metabolic zonation of liver parenchyma. Semin. Liver Dis..

[48] Carrero P., Okamoto K., Coumailleau P., O’Brien S., Tanaka H., Poellinger L. (2000). Redox-regulated recruitment of the transcriptional coactivators CREB-binding protein and SRC-1 to hypoxia-inducible factor 1α. Mol. Cell. Biol..

[49] Kallio P.J., Okamoto K., O’Brien S., Carrero P., Makino Y., Tanaka H., Poellinger L. (1998). Signal transduction in hypoxic cells: Inducible nuclear translocation and recruitment of the CBP/p300 coactivator by the hypoxia-inducible factor-1α. EMBO J..

[50] Diebold I., Flügel D., Becht S., Belaiba R.S., Bonello S., Hess J., Kietzmann T., Görlach A. (2010). The hypoxia-inducible factor-2α is stabilized by oxidative stress involving NOX4. Antioxid. Redox Signal..

[51] Brunelle J.K., Bell E.L., Quesada N.M., Vercauteren K., Tiranti V., Zeviani M., Scarpulla R.C., Chandel N.S. (2005). Oxygen sensing requires mitochondrial ROS but not oxidative phosphorylation. Cell Metab..

[52] Liu Q., Berchner-Pfannschmidt U., Moller U., Brecht M., Wotzlaw C., Acker H., Jungermann K., Kietzmann T. (2004). A Fenton reaction at the endoplasmic reticulum is involved in the redox control of hypoxia-inducible gene expression. Proc. Natl. Acad. Sci. USA.

[53] Bonello S., Zahringer C., BelAiba R.S., Djordjevic T., Hess J., Michiels C., Kietzmann T., Görlach A. (2007). Reactive oxygen species activate the HIF-1α promoter via a functional NF-κB site. Arterioscler. Thromb. Vasc. Biol..

[54] BelAiba R.S., Bonello S., Zahringer C., Schmidt S., Hess J., Kietzmann T., Görlach A. (2007). Hypoxia Up-Regulates Hypoxia-Inducible Factor-1α Transcription by Involving Phosphatidylinositol 3-Kinase and Nuclear Factor κB in Pulmonary Artery Smooth Muscle Cells. Mol. Biol. Cell.

[55] Rius J., Guma M., Schachtrup C., Akassoglou K., Zinkernagel A.S., Nizet V., Johnson R.S., Haddad G.G., Karin M. (2008). NF-κB links innate immunity to the hypoxic response through transcriptional regulation of HIF-1α. Nature.

[56] Görlach A., Bonello S. (2008). The cross-talk between NF-κB and HIF-1: Further evidence for a significant liaison. Biochem. J..

[57] Van Uden P., Kenneth N.S., Rocha S. (2008). Regulation of hypoxia-inducible factor-1αa by NF-κB. Biochem. J..

[58] Scholz C.C., Taylor C.T. (2013). Targeting the HIF pathway in inflammation and immunity. Curr. Opin. Pharmacol..

[59] Al Taleb Z., Petry A., Chi T.F., Mennerich D., Görlach A., Dimova E.Y., Kietzmann T. (2016). Differential transcriptional regulation of hypoxia-inducible factor-1a by arsenite under normoxia and hypoxia: Involvement of Nrf2. J. Mol. Med..

[60] Lacher S.E., Levings D.C., Freeman S., Slattery M. (2018). Identification of a functional antioxidant response element at the HIF1A locus. Redox Biol..

[61] Koong A.C., Chen E.Y., Mivechi N.F., Denko N.C., Stambrook P., Giaccia A.J. (1994). Hypoxic Activation of Nuclear Factor-kB Is Mediated by a Ras and Raf Signaling Pathway and Does Not Involve MAP Kinase (ERK1 or ERK2). Cancer Res..

[62] Nilakantan H., Kuttippurathu L., Parrish A., Hoek J.B., Vadigepalli R. (2015). In vivo zonal variation and liver cell-type specific NF-κB localization after chronic adaptation to ethanol and following partial hepatectomy. PLoS ONE.

[63] Skoko J.J., Wakabayashi N., Noda K., Kimura S., Tobita K., Shigemura N., Tsujita T., Yamamoto M., Kensler T.W. (2014). Loss of Nrf2 in mice evokes a congenital intrahepatic shunt that alters hepatic oxygen and protein expression gradients and toxicity. Toxicol. Sci..

[64] Varela-Nallar L., Rojas-Abalos M., Abbott A.C., Moya E.A., Iturriaga R., Inestrosa N.C. (2014). Chronic hypoxia induces the activation of the Wnt/β-catenin signaling pathway and stimulates hippocampal neurogenesis in wild-type and APPswe-PS1ΔE9 transgenic mice in vivo. Front. Cell. Neurosci..

[65] Mazumdar J., O’Brien W.T., Johnson R.S., Lamanna J.C., Chavez J.C., Klein P.S., Simon M.C. (2010). O2 regulates stem cells through Wnt/ß-catenin signalling. Nat. Cell Biol..

[66] Zhang Q., Lou Y., Zhang J., Fu Q., Wei T., Sun X., Chen Q., Yang J., Bai X., Liang T. (2017). Hypoxia-inducible factor-2Î± promotes tumor progression and has crosstalk with Wnt/Î²-catenin signaling in pancreatic cancer. Mol. Cancer.

[67] Kaidi A., Williams A.C., Paraskeva C. (2007). Interaction between ß-catenin and HIF-1 promotes cellular adaptation to hypoxia. Nat. Cell Biol..

[68] Verras M., Papandreou I., Lim A.L., Denko N.C. (2008). Tumor hypoxia blocks Wnt processing and secretion through the induction of endoplasmic reticulum stress. Mol. Cell. Biol..

[69] Näthke I., Rocha S. (2011). Antagonistic crosstalk between APC and HIF-1α. Cell Cycle.

[70] Newton I.P., Kenneth N.S., Appleton P.L., Näthke I., Rocha S. (2010). Adenomatous polyposis coli and hypoxia-inducible factor-1α have an antagonistic connection. Mol. Biol. Cell.

[71] Brocardo M., Lei Y., Tighe A., Taylor S.S., Mok M.T.S., Henderson B.R. (2008). Mitochondrial targeting of adenomatous polyposis coli protein is stimulated by truncating cancer mutations: Regulation of Bcl-2 and implications for cell survival. J. Biol. Chem..

[72] Lui C., Mills K., Brocardo M.G., Sharma M., Henderson B.R. (2012). APC as a mobile scaffold: Regulation and function at the nucleus, centrosomes, and mitochondria. IUBMB Life.

[73] Woo D.K., Green P.D., Santos J.H., D’Souza A.D., Walther Z., Martin W.D., Christian B.E., Chandel N.S., Shadel G.S. (2012). Mitochondrial genome instability and ROS enhance intestinal tumorigenesis in APC Min/+ mice. Am. J. Pathol..

[74] Hoogeboom D., Burgering B.M.T. (2009). Should I stay or should I go: β-catenin decides under stress. Biochim. Biophys. Acta Rev. Cancer.

[75] Görlach A., Dimova E.Y., Petry A., Martinez-Ruiz A., Hernansanz-Agustin P., Rolo A.P., Palmeira C.M., Kietzmann T. (2015). Reactive oxygen species, nutrition, hypoxia and diseases: Problems solved?. Redox Biol..

[76] Lenart J., Dombrowski F., Görlach A., Kietzmann T. (2007). Deficiency of manganese superoxide dismutase in hepatocytes disrupts zonated gene expression in mouse liver. Arch. Biochem. Biophys..

[77] Konzack A., Jakupovic M., Kubaichuk K., Görlach A., Dombrowski F., Miinalainen I., Sormunen R., Kietzmann T. (2015). Mitochondrial Dysfunction Due to Lack of Manganese Superoxide Dismutase Promotes Hepatocarcinogenesis. Antioxid. Redox Signal..

[78] Reed K.R., Athineos D., Meniel V.S., Wilkins J.A., Ridgway R.A., Burke Z.D., Muncan V., Clarke A.R., Sansom O.J. (2008). B-catenin deficiency, but not Myc deletion, suppresses the immediate phenotypes of APC loss in the liver. Proc. Natl. Acad. Sci. USA.

[79] Diehl A.M., Chute J. (2013). Underlying potential: Cellular and molecular determinants of adult liver repair. J. Clin. Investig..

[80] Michelotti G.A., Machado M.V., Diehl A.M. (2013). NAFLD, NASH and liver cancer. Nat. Rev. Gastroenterol. Hepatol..

[81] Sicklick J.K., Li Y.-X., Choi S.S., Qi Y., Chen W., Bustamante M., Huang J., Zdanowicz M., Camp T., Torbenson M.S. (2005). Role for Hedgehog signaling in hepatic stellate cell activation and viability. Lab. Investig..

[82] Omenetti A., Choi S., Michelotti G., Diehl A.M. (2011). Hedgehog signaling in the liver. J. Hepatol..

[83] Teperino R., Aberger F., Esterbauer H., Riobo N., Pospisilik J.A. (2014). Canonical and non-canonical hedgehog signalling and the control of metabolism. Semin. Cell Dev. Biol..

[84] Gebhardt R. (1992). Metabolic zonation of the liver: Regulation and implications for liver function. Pharmacol. Ther..

[85] Postic C., Girard J. (2008). The role of the lipogenic pathway in the development of hepatic steatosis. Diabetes Metab..

[86] Matz-Soja M., Rennert C., Schonefeld K., Aleithe S., Boettger J., Schmidt-Heck W., Weiss T.S., Hovhannisyan A., Zellmer S., Kloting N. (2016). Hedgehog signaling is a potent regulator of liver lipid metabolism and reveals a GLI-code associated with steatosis. Elife.

[87] Hazel S.J., Sandberg Nordqvist A., Hall K., Nilsson M., Schalling M. (1998). Differential expression of IGF-I and IGF-binding protein-1 and -2 in periportal and perivenous zones of rat liver. J. Endocrinol..

[88] Matz-Soja M., Aleithe S., Marbach E., Böttger J., Arnold K., Schmidt-Heck W., Kratzsch J., Gebhardt R. (2014). Hepatic Hedgehog signaling contributes to the regulation of IGF1 and IGFBP1 serum levels. Cell Commun. Signal..

[89] Bijlsma M.F., Groot A.P., Oduro J.P., Franken R.J., Schoenmakers S.H., Peppelenbosch M.P., Spek C.A. (2009). Hypoxia induces a hedgehog response mediated by HIF-1α. J. Cell. Mol. Med..

[90] Zhou J., Wu K., Gao D., Zhu G., Wu D., Wang X., Chen Y., Du Y., Song W., Ma Z. (2014). Reciprocal regulation of hypoxia-inducible factor 2α and GLI1 expression associated with the radioresistance of renal cell carcinoma. Int. J. Radiat. Oncol. Biol. Phys..

[91] Onishi H., Yamasaki A., Kawamoto M., Imaizumi A., Katano M. (2016). Hypoxia but not normoxia promotes Smoothened transcription through upregulation of RBPJ and Mastermind-like 3 in pancreatic cancer. Cancer Lett..

[92] Chen S., Zhang M., Xing L., Wang Y., Xiao Y., Wu Y. (2015). HIF-1a contributes to proliferation and invasiveness of neuroblastoma cells via SHH signaling. PLoS ONE.

[93] Furuta E., Pai S.K., Zhan R., Bandyopadhyay S., Watabe M., Mo Y.Y., Hirota S., Hosobe S., Tsukada T., Miura K. (2008). Fatty acid synthase gene is up-regulated by hypoxia via activation of Akt and sterol regulatory element binding protein-1. Cancer Res..

[94] Huang D., Li T., Li X., Zhang L., Sun L., He X., Zhong X., Jia D., Song L., Semenza G.L. (2014). HIF-1-Mediated Suppression of Acyl-CoA Dehydrogenases and Fatty Acid Oxidation Is Critical for Cancer Progression. Cell Rep..

[95] Yimlamai D., Christodoulou C., Galli G.G., Yanger K., Pepe-Mooney B., Gurung B., Shrestha K., Cahan P., Stanger B.Z., Camargo F.D. (2014). Hippo pathway activity influences liver cell fate. Cell.

[96] Fitamant J., Kottakis F., Benhamouche S., Tian H.S., Chuvin N., Parachoniak C.A., Nagle J.M., Perera R.M., Lapouge M., Deshpande V. (2015). YAP Inhibition Restores Hepatocyte Differentiation in Advanced HCC, Leading to Tumor Regression. Cell Rep..

[97] Font-Burgada J., Shalapour S., Ramaswamy S., Hsueh B., Rossell D., Umemura A., Taniguchi K., Nakagawa H., Valasek M.A., Ye L. (2015). Hybrid Periportal Hepatocytes Regenerate the Injured Liver without Giving Rise to Cancer. Cell.

[98] Lu L., Li Y., Kim S.M., Bossuyt W., Liu P., Qiu Q., Wang Y., Halder G., Finegold M.J., Lee J.S. (2010). Hippo signaling is a potent in vivo growth and tumor suppressor pathway in the mammalian liver. Proc. Natl. Acad. Sci. USA.

[99] Grijalva J.L., Huizenga M., Mueller K., Rodriguez S., Brazzo J., Camargo F., Sadri-Vakili G., Vakili K. (2014). Dynamic alterations in Hippo signaling pathway and YAP activation during liver regeneration. Am. J. Physiol. Gastrointest. Liver Physiol..

[100] Xiang L., Gilkes D.M., Hu H., Takano N., Luo W., Lu H., Bullen J.W., Samanta D., Liang H., Semenza G.L. (2014). Hypoxia-inducible factor 1 mediates TAZ expression and nuclear localization to induce the breast cancer stem cell phenotype. Oncotarget.

[101] Bendinelli P., Maroni P., Matteucci E., Luzzati A., Perrucchini G., Desiderio M.A. (2013). Hypoxia inducible factor-1 is activated by transcriptional co-activator with PDZ-binding motif (TAZ) versus WWdomain-containing oxidoreductase (WWOX) in hypoxic microenvironment of bone metastasis from breast cancer. Eur. J. Cancer.

[102] Xiang L., Gilkes D.M., Hu H., Luo W., Bullen J.W., Liang H., Semenza G.L. (2015). HIF-1α and TAZ serve as reciprocal co-activators in human breast cancer cells. Oncotarget.

[103] Zhang X., Li Y., Ma Y., Yang L., Wang T., Meng X., Zong Z., Sun X., Hua X., Li H. (2018). Yes-associated protein (YAP) binds to HIF-1α and sustains HIF-1α protein stability to promote hepatocellular carcinoma cell glycolysis under hypoxic stress. J. Exp. Clin. Cancer Res..

[104] Elpek G.O. (2015). Angiogenesis and liver fibrosis. World J. Hepatol..

[105] Zhang C., Bian M., Chen X., Jin H., Zhao S., Yang X., Shao J., Chen A., Guo Q., Zhang F. (2018). Oroxylin A prevents angiogenesis of LSECs in liver fibrosis via inhibition of YAP/HIF-1α signaling. J. Cell. Biochem..

[106] Zhang L., Wang Y.D., Chen W.D., Wang X., Lou G., Liu N., Lin M., Forman B.M., Huang W. (2012). Promotion of liver regeneration/repair by farnesoid X receptor in both liver and intestine in mice. Hepatology.

[107] Kim W., Khan S.K., Gvozdenovic-Jeremic J., Kim Y., Dahlman J., Kim H., Park O., Ishitani T., Jho E.H., Gao B. (2017). Hippo signaling interactions with Wnt/β-catenin and Notch signaling repress liver tumorigenesis. J. Clin. Investig..

[108] Pear W.S., Simon M.C. (2005). Lasting longer without oxygen: The influence of hypoxia on Notch signaling. Cancer Cell.

[109] Szczepaniak L.S., Nurenberg P., Leonard D., Browning J.D., Reingold J.S., Grundy S., Hobbs H.H., Dobbins R.L. (2005). Magnetic resonance spectroscopy to measure hepatic triglyceride content: Prevalence of hepatic steatosis in the general population. Am. J. Physiol. Endocrinol. Metab..

[110] Younossi Z., Anstee Q.M., Marietti M., Hardy T., Henry L., Eslam M., George J., Bugianesi E. (2018). Global burden of NAFLD and NASH: Trends, predictions, risk factors and prevention. Nat. Rev. Gastroenterol. Hepatol..

[111] Dowman J.K., Tomlinson J.W., Newsome P.N. (2011). Systematic review: The diagnosis and staging of non-alcoholic fatty liver disease and non-alcoholic steatohepatitis. Aliment. Pharmacol. Ther..

[112] Anstee Q.M., Targher G., Day C.P. (2013). Progression of NAFLD to diabetes mellitus, cardiovascular disease or cirrhosis. Nat. Rev. Gastroenterol. Hepatol..

[113] Argo C.K., Caldwell S.H. (2009). Epidemiology and Natural History of Non-Alcoholic Steatohepatitis. Clin. Liver Dis..

[114] Hui J.M., Kench J.G., Chitturi S., Sud A., Farrell G.C., Byth K., Hall P., Khan M., George J. (2003). Long-term outcomes of cirrhosis in nonalcoholic steatohepatitis compared with hepatitis C. Hepatology.

[115] Adams L.A., Lymp J.F., St. Sauver J., Sanderson S.O., Lindor K.D., Feldstein A., Angulo P. (2005). The natural history of nonalcoholic fatty liver disease: A population-based cohort study. Gastroenterology.

[116] Tilg H., Moschen A.R. (2010). Evolution of inflammation in nonalcoholic fatty liver disease: The multiple parallel hits hypothesis. Hepatology.

[117] Yki-Jarvinen H. (2014). Non-alcoholic fatty liver disease as a cause and a consequence of metabolic syndrome. Lancet Diabetes Endocrinol..

[118] Kondo K., Sugioka T., Tsukada K., Aizawa M., Takizawa M., Shimizu K., Morimoto M., Suematsu M., Goda N. (2010). Fenofibrate, a peroxisome proliferator-activated receptor a agonist, improves hepatic microcirculatory patency and oxygen availability in a high-fat-diet-induced fatty liver in mice. Oxygen Transport to Tissue XXXI.

[119] Piguet A.-C., Stroka D., Zimmermann A., Dufour J.-F. (2010). Hypoxia aggravates non-alcoholic steatohepatitis in mice lacking hepatocellular PTEN. Clin. Sci..

[120] Schleicher J., Guthke R., Dahmen U., Dirsch O., Holzhuetter H.G., Schuster S. (2014). A theoretical study of lipid accumulation in the liver—Implications for nonalcoholic fatty liver disease. Biochim. Biophys. Acta Mol. Cell. Biol. Lipids.

[121] Quistorff B., Katz N., Witters L.A. (1992). Hepatocyte heterogeneity in the metabolism of fatty acids: Discrepancies on zonation of acetyl-CoA carboxylase. Enzyme.

[122] Debois D., Bralet M.-P., Le Naour F., Brunelle A., Laprévote O. (2009). In Situ lipidomic analysis of nonalcoholic fatty liver by cluster TOF-SIMS imaging. Anal. Chem..

[123] Wattacheril J., Seeley E.H., Angel P., Chen H., Bowen B.P., Lanciault C., Caprioli R.M., Abumrad N., Flynn C.R. (2013). Differential Intrahepatic Phospholipid Zonation in Simple Steatosis and Nonalcoholic Steatohepatitis. PLoS ONE.

[124] Kim W.Y., Safran M., Buckley M.R., Ebert B.L., Glickman J., Bosenberg M., Regan M., Kaelin W.G. (2006). Failure to prolyl hydroxylate hypoxia-inducible factor α phenocopies VHL inactivation in vivo. EMBO J..

[125] Walter K.M., Schonenberger M.J., Trotzmuller M., Horn M., Elsasser H.P., Moser A.B., Lucas M.S., Schwarz T., Gerber P.A., Faust P.L. (2014). Hif-2α promotes degradation of mammalian peroxisomes by selective autophagy. Cell Metab..

[126] Farr R.L., Lismont C., Terlecky S.R., Fransen M. (2016). Peroxisome biogenesis in mammalian cells: The impact of genes and environment. Biochim. Biophys. Acta.

[127] Zhou J., Zhang S., Xue J., Avery J., Wu J., Lind S.E., Ding W.Q. (2012). Activation of peroxisome proliferator-activated receptor α (PPARα) suppresses hypoxia-inducible factor-1α (HIF-1α) signaling in cancer cells. J. Biol. Chem..

[128] Puri P., Wiest M.M., Cheung O., Mirshahi F., Sargeant C., Min H.K., Contos M.J., Sterling R.K., Fuchs M., Zhou H. (2009). The plasma lipidomic signature of nonalcoholic steatohepatitis. Hepatology.

[129] Morello E., Sutti S., Foglia B., Novo E., Cannito S., Bocca C., Rajsky M., Bruzzì S., Abate M.L., Rosso C. (2018). Hypoxia-inducible factor 2a drives nonalcoholic fatty liver progression by triggering hepatocyte release of histidine-rich glycoprotein. Hepatology.

[130] Xie C., Yagai T., Luo Y., Liang X., Chen T., Wang Q., Sun D., Zhao J., Ramakrishnan S.K., Sun L. (2017). Activation of intestinal hypoxia-inducible factor 2α during obesity contributes to hepatic steatosis. Nat. Med..

[131] Kaelin W.G., Ratcliffe P.J. (2008). Oxygen Sensing by Metazoans: The Central Role of the HIF Hydroxylase Pathway. Mol. Cell.

[132] Kim J.W., Tchernyshyov I., Semenza G.L., Dang C.V. (2006). HIF-1-mediated expression of pyruvate dehydrogenase kinase: A metabolic switch required for cellular adaptation to hypoxia. Cell Metab..

[133] Lu C.-W., Lin S.-C., Chen K.-F., Lai Y.-Y., Tsai S.-J. (2008). Induction of pyruvate dehydrogenase kinase-3 by hypoxia-inducible factor-1 promotes metabolic switch and drug resistance. J. Biol. Chem..

[134] Tello D., Balsa E., Acosta-Iborra B., Fuertes-Yebra E., Elorza A., Ordóñez A., Corral-Escariz M., Soro I., López-Bernardo E., Perales-Clemente E. (2011). Induction of the mitochondrial NDUFA4L2 protein by HIF-1α decreases oxygen consumption by inhibiting complex i activity. Cell Metab..

[135] Fukuda R., Zhang H., Kim J.W., Shimoda L., Dang C.V., Semenza G.L. (2007). HIF-1 regulates cytochrome oxidase subunits to optimize efficiency of respiration in hypoxic cells. Cell.

[136] Zhang H., Bosch-Marce M., Shimoda L.A., Tan Y.S., Baek J.H., Wesley J.B., Gonzalez F.J., Semenza G.L. (2008). Mitochondrial autophagy is an HIF-1-dependent adaptive metabolic response to hypoxia. J. Biol. Chem..

[137] Chan S.Y., Zhang Y.Y., Hemann C., Mahoney C.E., Zweier J.L., Loscalzo J. (2009). MicroRNA-210 Controls Mitochondrial Metabolism during Hypoxia by Repressing the Iron-Sulfur Cluster Assembly Proteins ISCU1/2. Cell Metab..

[138] Naveau S., Giraud V., Borotto E., Aubert A., Capron F., Chaput J. (1997). Excess weight risk factor for alcoholic liver disease. Hepatology.

[139] Gao B., Bataller R. (2011). Alcoholic liver disease: Pathogenesis and new therapeutic targets. Gastroenterology.

[140] Siu L., Foont J., Wands J.R. (2009). Hepatitis C virus and alcohol. Semin. Liver Dis..

[141] O’Shea R.S., Dasarathy S., McCullough A.J., Shuhart M.C., Davis G.L., Franco J., Harrison S.A., Howell C.D., Ling S.C., Liu L.U. (2010). Alcoholic liver disease. Hepatology.

[142] Mandayam S., Jamal M.M., Morgan T.R. (2004). Epidemiology of alcoholic liver disease. Semin. Liver Dis..

[143] Israel Y., Videla L., MacDonald A., Bernstein J. (1973). Metabolic alterations produced in the liver by chronic ethanol administration. Comparison between the effects produced by ethanol and by thyroid hormones. Biochem. J..

[144] Yuki T., Thurman R.G. (1980). The swift increase in alcohol metabolism: Time course for the increase in hepatic oxygen uptake and the involvement of glycolysis. Biochem. J..

[145] French S.W. (2004). The role of hypoxia in the pathogenesis of alcoholic liver disease. Hepatol. Res..

[146] Jungermann K., Kietzmann T. (2000). Oxygen: Modulator of metabolic zonation and disease of the liver. Hepatology.

[147] Arteel G.E., Raleigh J.A., Bradford B.U., Thurman R.G., Hara H. (1996). Acute alcohol produces hypoxia directly in rat liver tissue in vivo: Role of Kupffer cells. Am. J. Physiol. Gastrointest. Liver Physiol..

[148] Arteel G.E., Iimuro Y., Yin M., Raleigh J.A., Thurman R.G. (1997). Chronic enteral ethanol treatment causes hypoxia in rat liver tissue in vivo. Hepatology.

[149] Nath B., Levin I., Csak T., Petrasek J., Mueller C., Kodys K., Catalano D., Mandrekar P., Szabo G. (2011). Hepatocyte-specific hypoxia-inducible factor-1α is a determinant of lipid accumulation and liver injury in alcohol-induced steatosis in mice. Hepatology.

[150] Nishiyama Y., Goda N., Kanai M., Niwa D., Osanai K., Yamamoto Y., Senoo-Matsuda N., Johnson R.S., Miura S., Kabe Y. (2012). HIF-1α induction suppresses excessive lipid accumulation in alcoholic fatty liver in mice. J. Hepatol..

[151] Laitakari A., Ollonen T., Kietzmann T., Walkinshaw G., Mennerich D., Izzi V., Haapasaari K.M., Myllyharju J., Serpi R., Dimova E.Y. (2019). Systemic inactivation of hypoxia-inducible factor prolyl 4-hydroxylase 2 in mice protects from alcohol-induced fatty liver disease. Redox Biol..

[152] Richter K., Konzack A., Pihlajaniemi T., Heljasvaara R., Kietzmann T. (2015). Redox-fibrosis: Impact of TGFβ1 on ROS generators, mediators and functional consequences. Redox Biol..

[153] Novo E., Povero D., Busletta C., Paternostro C., Di Bonzo L.V., Cannito S., Compagnone A., Bandino A., Marra F., Colombatto S. (2012). The biphasic nature of hypoxia-induced directional migration of activated human hepatic stellate cells. J. Pathol..

[154] Moon J.O., Welch T.P., Gonzalez F.J., Copple B.L. (2009). Reduced liver fibrosis in hypoxia-inducible factor-1α-deficient mice. Am. J. Physiol. Gastrointest. Liver Physiol..

[155] Roychowdhury S., Chiang D.J., McMullen M.R., Nagy L.E. (2014). Moderate, chronic ethanol feeding exacerbates carbon tetrachloride–induced hepatic fibrosis via hepatocyte-specific hypoxia-inducible factor 1α. Pharmacol. Res. Perspect..

[156] Copple B.L., Kaska S., Wentling C. (2012). Hypoxia-inducible factor activation in myeloid cells contributes to the development of liver fibrosis in cholestatic mice. J. Pharmacol. Exp. Ther..

[157] Copple B.L., Bai S., Burgoon L.D., Moon J.-O. (2011). Hypoxia-inducible factor-1α regulates the expression of genes in hypoxic hepatic stellate cells important for collagen deposition and angiogenesis. Liver Int..

[158] Zagzag D., Krishnamachary B., Yee H., Okuyama H., Chiriboga L., Ali M.A., Melamed J., Semenza G.L. (2005). Stromal cell-derived factor-1α and CXCR4 expression in hemangioblastoma and clear cell-renal cell carcinoma: Von Hippel-Lindau loss-of-function induces expression of a ligand and its receptor. Cancer Res..

[159] Hempel M., Schmitz A., Winkler S., Kucukoglu O., Bruckner S., Niessen C., Christ B. (2015). Pathological implications of cadherin zonation in mouse liver. Cell. Mol. Life Sci..

[160] Rowe R.G., Lin Y., Shimizu-Hirota R., Hanada S., Neilson E.G., Greenson J.K., Weiss S.J. (2011). Hepatocyte-derived Snail1 propagates liver fibrosis progression. Mol. Cell. Biol..

[161] Cicchini C., Amicone L., Alonzi T., Marchetti A., Mancone C., Tripodi M. (2015). Molecular mechanisms controlling the phenotype and the EMT/MET dynamics of hepatocyte. Liver Int..

[162] Liu Y., Liu Y., Yan X., Xu Y., Luo F., Ye J., Yan H., Yang X., Huang X., Zhang J. (2014). HIFs enhance the migratory and neoplastic capacities of hepatocellular carcinoma cells by promoting EMT. Tumour Biol..

[163] Krishnamachary B., Zagzag D., Nagasawa H., Rainey K., Okuyama H., Baek J.H., Semenza G.L. (2006). Hypoxia-inducible factor-1-dependent repression of E-cadherin in von Hippel-Lindau tumor suppressor-null renal cell carcinoma mediated by TCF3, ZFHX1A, and ZFHX1B. Cancer Res..

[164] Yang M.H., Wu M.Z., Chiou S.H., Chen P.M., Chang S.Y., Liu C.J., Teng S.C., Wu K.J. (2008). Direct regulation of TWIST by HIF-1α promotes metastasis. Nat. Cell Biol..

[165] Liu Y., Jiang L., Sun C., Ireland N., Shah Y.M., Liu Y., Rui L. (2018). Insulin/Snail1 axis ameliorates fatty liver disease by epigenetically suppressing lipogenesis. Nat. Commun..

[166] Torre L.A., Bray F., Siegel R.L., Ferlay J., Lortet-Tieulent J., Jemal A. (2015). Global cancer statistics, 2012. CA Cancer J. Clin..

[167] Jemal A., Bray F., Center M.M., Ferlay J., Ward E., Forman D. (2011). Global cancer statistics. CA Cancer J. Clin..

[168] Llovet J.M., Montal R., Sia D., Finn R.S. (2018). Molecular therapies and precision medicine for hepatocellular carcinoma. Nat. Rev. Clin. Oncol..

[169] Guo X., Li D., Chen Y., An J., Wang K., Xu Z., Chen Z., Xing J. (2015). SNP rs2057482 in HIF1A gene predicts clinical outcome of aggressive hepatocellular carcinoma patients after surgery. Sci. Rep..

[170] Hsiao P.C., Chen M.K., Su S.C., Ueng K.C., Chen Y.C., Hsieh Y.H., Liu Y.F., Tsai H.T., Yang S.F. (2010). Hypoxia inducible factor-1α gene polymorphism G1790A and its interaction with tobacco and alcohol consumptions increase susceptibility to hepatocellular carcinoma. J. Surg. Oncol..

[171] Li S., Yao D., Wang L., Wu W., Qiu L., Yao M., Yao N., Zhang H., Yu D., Ni Q. (2011). Expression characteristics of hypoxia-inducible factor-1α and its clinical values in diagnosis and prognosis of hepatocellular carcinoma. Hepatitis Mon..

[172] Bangoura G., Liu Z.S., Qian Q., Jiang C.Q., Yang G.F., Jing S. (2007). Prognostic significance of HIF-2α/EPAS1 expression in hepatocellular carcinoma. World J. Gastroenterol..

[173] Xie H., Song J., Liu K., Ji H., Shen H., Hu S., Yang G., Du Y., Zou X., Jin H. (2008). The expression of hypoxia-inducible factor-1α in hepatitis B virus-related hepatocellular carcinoma: Correlation with patients’ prognosis and hepatitis B virus X protein. Dig. Dis. Sci..

[174] Simon F., Bockhorn M., Praha C., Baba H.A., Broelsch C.E., Frilling A., Weber F. (2010). Deregulation of HIF1-α and hypoxia-regulated pathways in hepatocellular carcinoma and corresponding non-malignant liver tissue-influence of a modulated host stroma on the prognosis of HCC. Langenbeck’s Arch. Surg..

[175] Xiang Z.L., Zeng Z.C., Fan J., Tang Z.Y., He J., Zeng H.Y., Chang J.Y. (2012). The expression of HIF-1α in primary hepatocellular carcinoma and its correlation with radiotherapy response and clinical outcome. Mol. Biol. Rep..

[176] Yang S.L., Liu L.P., Jiang J.X., Xiong Z.F., He Q.J., Wu C. (2014). The correlation of expression levels of HIF-1α and HIF-2α in hepatocellular carcinoma with capsular invasion, portal vein tumor thrombi and patients’ clinical outcome. Jpn. J. Clin. Oncol..

[177] Zheng S.S., Chen X.H., Yin X., Zhang B.H. (2013). Prognostic Significance of HIF-1α Expression in Hepatocellular Carcinoma: A Meta-Analysis. PLoS ONE.

[178] Wilson G.K., Brimacombe C.L., Rowe I.A., Reynolds G.M., Fletcher N.F., Stamataki Z., Bhogal R.H., Simões M.L., Ashcroft M., Afford S.C. (2012). A dual role for hypoxia inducible factor-1α in the hepatitis C virus lifecycle and hepatoma migration. J. Hepatol..

[179] Sun H.X., Xu Y., Yang X.R., Wang W.M., Bai H., Shi R.Y., Nayar S.K., Devbhandari R.P., He Y.Z., Zhu Q.F. (2013). Hypoxia inducible factor 2 α inhibits hepatocellular carcinoma growth through the transcription factor dimerization partner 3/E2F transcription factor 1-dependent apoptotic pathway. Hepatology.

[180] Rey S., Semenza G.L. (2010). Hypoxia-inducible factor-1-dependent mechanisms of vascularization and vascular remodelling. Cardiovasc. Res..

[181] Zhen L., Shijie N., Shuijun Z. (2014). Tumor PHD2 expression is correlated with clinical features and prognosis of patients with HCC receiving liver resection. Medicine.

[182] Tao Y., Lin F., Li R., Shen J., Wang Z. (2016). Prolyl hydroxylase-2 inhibits liver tumor cell proliferation and cyclin D1 expression in a hydroxylase-dependent manner. Int. J. Biochem. Cell Biol..

[183] Jiang L., Liu Y., Ma C., Li B. (2018). MicroRNA-30a suppresses the proliferation, migration and invasion of human renal cell carcinoma cells by directly targeting ADAM9. Oncol. Lett..

[184] Ma M., Hua S., Li G., Wang S., Cheng X., He S., Wu P., Chen X. (2017). Prolyl hydroxylase domain protein 3 and asparaginyl hydroxylase factor inhibiting HIF-1 levels are predictive of tumoral behavior and prognosis in hepatocellular carcinoma. Oncotarget.

[185] Mazzone M., Dettori D., de Oliveira R.L., Loges S., Schmidt T., Jonckx B., Tian Y.M., Lanahan A.A., Pollard P., de Almodovar C.R. (2009). Heterozygous deficiency of PHD2 restores tumor oxygenation and inhibits metastasis via endothelial normalization. Cell.

[186] Harnoss J.M., Platzer L.K., Burhenne J., Radhakrishnan P., Cai J., Strowitzki M.J., Weiss J., Ritter A.S., Mollenhauer M., Schmidt T. (2017). Prolyl Hydroxylase Inhibition Enhances Liver Regeneration Without Induction of Tumor Growth. Ann. Surg..

[187] Van de Groenendaal-Meent D., Adel M.D., Noukens J., Rijnders S., Krebs-Brown A., Mateva L., Alexiev A., Schaddelee M. (2016). Effect of Moderate Hepatic Impairment on the Pharmacokinetics and Pharmacodynamics of Roxadustat, an Oral Hypoxia-Inducible Factor Prolyl Hydroxylase Inhibitor. Clin. Drug Investig..

[188] Howard T.K., Klintmalm G.B.G., Cofer J.B., Husberg B.S., Goldstein R.M., Gonwa T.A. (1990). The influence of preservation injury on rejection in the hepatic transplant recipient. Transplantation.

[189] Serracino-Inglott F., Habib N.A., Mathie R.T. (2001). Hepatic ischemia-reperfusion injury. Am. J. Surg..

[190] Guo J.Y., Yang T., Sun X.G., Zhou N.Y., Li F.S., Long D., Lin T., Li P.Y., Feng L. (2011). Ischemic postconditioning attenuates liver warm ischemia-reperfusion injury through Akt-eNOS-NO-HIF pathway. J. Biomed. Sci..

[191] Plock J., Frese S., Keogh A., Bisch-Knaden S., Ayuni E., Corazza N., Weikert C., Jakob S., Erni D., Dufour J.-F. (2007). Activation of non-ischemic, hypoxia-inducible signalling pathways up-regulate cytoprotective genes in the murine liver. J. Hepatol..

[192] Zhong Z., Ramshesh V.K., Rehman H., Currin R.T., Sridharan V., Theruvath T.P., Kim I., Wright G.L., Lemasters J.J. (2008). Activation of the oxygen-sensing signal cascade prevents mitochondrial injury after mouse liver ischemia-reperfusion. Am. J. Physiol. Gastrointest. Liver Physiol..

[193] Ben Mosbah I., Mouchel Y., Pajaud J., Ribault C., Lucas C., Laurent A., Boudjema K., Morel F., Corlu A., Compagnon P. (2012). Pretreatment with Mangafodipir Improves Liver Graft Tolerance to Ischemia/Reperfusion Injury in Rat. PLoS ONE.

[194] Yang Y.Y., Lee P.C., Huang Y.T., Lee W.P., Kuo Y.J., Lee K.C., Hsieh Y.C., Lee T.Y., Lin H.C. (2014). Involvement of the HIF-1α and wnt/β-catenin pathways in the protective effects of losartan on fatty liver graft with ischaemia/reperfusion injury. Clin. Sci..

[195] Schneider M., Van Geyte K., Fraisl P., Kiss J., Aragonés J., Mazzone M., Mairbäurl H., De Bock K., Jeoung N.H., Mollenhauer M. (2010). Loss or Silencing of the PHD1 Prolyl Hydroxylase Protects Livers of Mice Against Ischemia/Reperfusion Injury. Gastroenterology.

[196] Eltzschig H.K., Eckle T. (2011). Ischemia and reperfusion-from mechanism to translation. Nat. Med..

[197] Fitzpatrick S.F., Fabian Z., Schaible B., Lenihan C.R., Schwarzl T., Rodriguez J., Zheng X., Li Z., Tambuwala M.M., Higgins D.G. (2016). Prolyl hydroxylase-1 regulates hepatocyte apoptosis in an NF-κB-dependent manner. Biochem. Biophys. Res. Commun..

[198] Mollenhauer M., Kiss J., Dudda J., Kirchberg J., Rahbari N., Radhakrishnan P., Niemietz T., Rausch V., Weitz J., Schneider M. (2012). Deficiency of the oxygen sensor PHD1 augments liver regeneration after partial hepatectomy. Langenbeck’s Arch. Surg..

[199] Adluri R.S., Thirunavukkarasu M., Dunna N.R., Zhan L., Oriowo B., Takeda K., Sanchez J.A., Otani H., Maulik G., Fong G.H. (2011). Disruption of hypoxia-inducible transcription factor-prolyl hydroxylase domain-1 (PHD-1-/-) attenuates ex vivo myocardial ischemia/reperfusion injury through hypoxia-inducible factor-1α transcription factor and its target genes in mice. Antioxid. Redox Signal..

[200] Hyvarinen J., Hassinen I.E., Sormunen R., Maki J.M., Kivirikko K.I., Koivunen P., Myllyharju J. (2010). Hearts of hypoxia-inducible factor prolyl 4-hydroxylase-2 hypomorphic mice show protection against acute ischemia-reperfusion injury. J. Biol. Chem..

[201] Karsikas S., Myllymäki M., Heikkilä M., Sormunen R., Kivirikko K.I., Myllyharju J., Serpi R., Koivunen P. (2016). HIF-P4H-2 deficiency protects against skeletal muscle ischemia-reperfusion injury. J. Mol. Med..

[202] Xie L., Pi X., Wang Z., He J., Willis M.S., Patterson C. (2015). Depletion of PHD3 protects heart from ischemia/reperfusion injury by inhibiting cardiomyocyte apoptosis. J. Mol. Cell. Cardiol..

[203] Cai Z., Luo W., Zhan H., Semenza G.L. (2013). Hypoxia-inducible factor 1 is required for remote ischemic preconditioning of the heart. Proc. Natl. Acad. Sci. USA.

[204] Sarkar K., Cai Z., Gupta R., Parajuli N., Fox-Talbot K., Darshan M.S., Gonzalez F.J., Semenza G.L. (2012). Hypoxia-inducible factor 1 transcriptional activity in endothelial cells is required for acute phase cardioprotection induced by ischemic preconditioning. Proc. Natl. Acad. Sci. USA.

[205] Kapitsinou P.P., Sano H., Michael M., Kobayashi H., Davidoff O., Bian A., Yao B., Zhang M.Z., Harris R.C., Duffy K.J. (2014). Endothelial HIF-2 mediates protection and recovery from ischemic kidney injury. J. Clin. Investig..

[206] Koeppen M., Lee J.W., Seo S.W., Brodsky K.S., Kreth S., Yang I.V., Buttrick P.M., Eckle T., Eltzschig H.K. (2018). Hypoxia-inducible factor 2-α-dependent induction of amphiregulin dampens myocardial ischemia-reperfusion injury. Nat. Commun..

[207] Olenchock B.A., Moslehi J., Baik A.H., Davidson S.M., Williams J., Gibson W.J., Chakraborty A.A., Pierce K.A., Miller C.M., Hanse E.A. (2016). EGLN1 Inhibition and Rerouting of α-Ketoglutarate Suffice for Remote Ischemic Protection. Cell.

[208] FibroGen FibroGen Announces Approval of Roxadustat in China for the Treatment of Anemia in Chronic Kidney Disease Patients on Dialysis [Media Release]. http://investor.fibrogen.com/phoenix.zhtml?c=253783&p=irol-newsArticle&ID=2380952.

[209] European Association for the Study of the Liver (2018). Electronic address: Easloffice@easloffice.eu, European Association for the Study of the Liver. EASL Clinical Practice Guidelines: Management of hepatocellular carcinoma. J. Hepatol..

